# Considering best practice standards for routine whole-genome sequencing for TB care and control

**DOI:** 10.5588/ijtldopen.24.0320

**Published:** 2024-10-01

**Authors:** J.T. Denholm, G. de Vries, R. Anthony, E. Robinson, M. Backx, I.F. Laurenson, A.L. Seagar, H. Modestil, L. Trieu, J.S. Meissner, D.H.L. Ng, J.Y. Tay, H.H. Lin, R. Lee, V. Sintchenko, B.J. Marais, E.J. Donnan

**Affiliations:** ^1^ Victorian Tuberculosis Program, Melbourne, VIC, Australia;; ^2^ Department of Infectious Diseases, Peter Doherty Institute for Infection and Immunity, University of Melbourne, Parkville, VIC, Australia;; ^3^ Centre for Infectious Disease Control, National Institute for Public Health and the Environment (RIVM), Bilthoven, The Netherlands;; ^4^ TB Unit and National Mycobacterial Reference Service, United Kingdom Health Security Agency, London, UK;; ^5^ Department of Microbiology, University Hospital of Wales, Cardiff, UK;; ^6^ Scottish Mycobacteria Reference Laboratory, Royal Infirmary of Edinburgh, Edinburgh, Scotland, UK;; ^7^ New York City Department of Health and Mental Hygiene, New York, NY, USA;; ^8^ National Tuberculosis Programme, National Centre for Infectious Diseases, Singapore;; ^9^ Institute of Epidemiology and Preventive Medicine, National Taiwan University College of Public Health, Taiwan;; ^10^ Faculty of Medicine and Health Sciences, McGill University, Montreal, QC, Canada;; ^11^ NSW Mycobacterium Reference Laboratory, Institute of Clinical Pathology and Medical Research, NSW Health, Sydney, NSW, Australia;; ^12^ Sydney Infectious Diseases Institute, The University of Sydney, Sydney, NSW, Australia;; ^13^ New South Wales Tuberculosis Program, Health Protection New South Wales, Sydney, NSW, Australia.

**Keywords:** *Mycobacterium tuberculosis*, tuberculosis, genomics, public health, computational biology, cluster analysis, policy making

## Abstract

TB is a priority pathogen for the application of whole-genome sequencing (WGS) into routine public health practice. In low-incidence settings, a growing number of services have begun to incorporate routine WGS into standard practice. The increasing availability of real-time genomic information supports a variety of aspects of the public health response, including the detection of drug resistance, monitoring of laboratory and clinical practices, contact tracing investigations and active case finding. Optimal structures and approaches are needed to support the rapid translation of genomic information into practice and to evaluate outcomes and impact. In this consensus paper, we outline the elements needed to systemically incorporate routine WGS into the TB public health response, including the sustainability of services, multidisciplinary team models and monitoring and evaluation frameworks. If integrated in an efficient and thoughtful manner, routine WGS has the potential to significantly improve clinical TB care for individuals and the overall public health response.

## RÉSUMÉ

La TB est un agent pathogène prioritaire pour l'application du séquençage du génome entier (WGS, pour l’anglais « *whole-genome sequencing* ») dans les pratiques courantes de santé publique. Dans des contextes à faible incidence, un nombre croissant de services ont commencé à intégrer le WGS de manière routinière dans la pratique standard. La disponibilité croissante de l'information génomique en temps réel soutient divers aspects de l'intervention de santé publique, notamment la détection de la résistance aux médicaments, la surveillance des pratiques de laboratoire et cliniques, les enquêtes de recherche des contacts et la recherche active des cas. Des structures et des approches optimales sont nécessaires pour soutenir l'application rapide de l'information génomique dans la pratique et pour évaluer les résultats et l'impact. Dans ce document de consensus, nous décrivons les éléments nécessaires à l'intégration systématique du WGS dans la riposte de santé publique à la TB, notamment la durabilité des services, les modèles d'équipe multidisciplinaire et les cadres de suivi et d'évaluation. S'il est intégré de manière efficace et réfléchie, le WGS de routine a le potentiel d'améliorer considérablement les soins cliniques de la TB pour les individus et la réponse globale de la santé publique.

The use of whole-genome sequencing (WGS) for the identification and surveillance of infectious diseases is increasingly routine in many settings. TB is regarded as a priority pathogen for the integration of WGS into routine practice, and initial systematic use of TB WGS has emphasised its added clinical value in the rapid diagnosis and identification of drug resistance, and prediction of susceptibility.^1–3^ While validation and implementation of genotypic drug resistance prediction is ongoing, in the majority of cases approaches are sufficiently validated for WGS to replace phenotypic drug susceptibility testing (DST) for first-line drugs.^4,5^ Existing WHO guidelines recommend genomic testing for rapid drug resistance assessment, such as next-generation sequencing.^6^ As WGS can also evaluate TB strain relatedness (including strain clustering and transmission inference), it offers additional benefits for public health. This capability is accessible from routine WGS but has additional complex analytic and translational requirements. Population-level TB analyses using WGS have provided important insights into the emergence of drug resistance and risk factors for transmission in various settings.^7,8^ Frequently, such analyses have been undertaken retrospectively, which limits the benefits for individual patient care and targeted programmatic response. However, the characteristics of TB (including long latency periods allowing opportunities for intervention and the prolonged nature of disease and treatment) mean that WGS and real time analysis may help guide and optimise public health interventions.

In recent years, a number of TB programmes have incorporated routine WGS and real-time analysis into patient care and public health activities.^9–11^ Previous work has identified a lack of standardisation hampering the widespread programmatic implementation of WGS and a need for harmonised approaches to report, monitor and evaluate impact.^12,13^ Following a series of joint programmatic reviews, semi-structured interviews and exchange visits conducted in 2023, we present a consensus view on real-time use of routine TB WGS. This is intended to support programmes contemplating the establishment of such services and to scope key elements for harmonising future practice.

## Operations and sustainability

Public health WGS programmes have been established using various models for operation and funding, often depending on research project grants with variation in terms of the sustainability of services.^14^ For systematic use at the programme level, this funding must be secure. It is also key to have access to a well validated, robust data pipeline(s) that is resilient and backed up as new information becomes available. This should include newly recognised mycobacterial identification, sensitivity and cluster information. Maintaining, validating and updating a pipeline to predict susceptibility and accurately genotype is complex and demanding, and is best performed where there is sufficient sample throughput and expert knowledge to justify and sustain the investment. In the United Kingdom, a common bioinformatic pipeline (COMPASS) has been developed to support common standards across neighbouring jurisdictions and laboratories, supporting harmonisation and the sharing of clustering and drug resistance information.^15^

Ideally, a robust programme should be developed to create cluster trees that show single nucleotide polymorphism (SNP) differences. Long-term sustainability is important for TB programmes in general, but confidence in long-term funding for genomic programmes is essential due to the need to curate mycobacterial isolates and genomic databases over extended periods of time. Although many services have been established with research grants (or other short-term project funds), funding for genomic testing, analysis, implementation and evaluation should be integrated into long-term funding models for TB programmes.

Multidisciplinary teams should be used to oversee WGS findings and support the implementation of results into clinical and public health practice. The optimal membership of such teams balances size and expertise, but diversity of personal and professional backgrounds is recognised as a high priority for effective engagement. Teams should include members with expertise in clinical medicine, field epidemiology, laboratory mycobacteriology and genomics, bioinformatics and health policy. Other disciplines, including social work, ethics and context-specific cultural workers, have additional value in the interpretation and integration of WGS into TB public health responses. Our respective teams meet regularly (typically weekly or fortnightly) for cluster review, with additional meetings coordinated for outbreaks of special significance. Beyond the local context, TB public health programmes integrating WGS should participate in regular inter-jurisdictional discussions about policy and practice. Such meetings, both formal and informal, allow opportunities for mutual support, quality assurance and dissemination of effective approaches, as well as case- and scenario-based reflection on emerging trends and novel experiences.

We also consider it critical that programmes actively involve members of TB-affected communities in culturally sensitive public health responses. This may take a variety of forms in different settings, but we would emphasise the importance of involving community members in evaluating genomic data rather than limiting their involvement to planning or evaluating specific public health activities.

In practical terms, it is most helpful to gather all available epidemiological data (including additional interviews where required) and rapidly convene multidisciplinary review to discuss any cluster where genomic links are suggested, but where the epidemiological connection is not immediately apparent (e.g., where a secondary case is a household member already identified). These meetings review what is known regarding the social and geographical context of cases and consider additional public health investigations, which may be helpful in understanding possible transmission links. Alternative explanations for apparent clusters, such as laboratory contamination events, may also be considered. Where cases with recognised connections are sequentially added to clusters over time, review meetings should be convened regularly to consider the overall epidemiological and social context, and to discuss the need for additional public health interventions.

## Models of translation

Performing and validating WGS and the closely related bioinformatic analysis are core service requirements for all programmes (Table 1). Historically, most services engaged in routine TB WGS have first developed pathways for reporting sequencing results to clinicians, mainly focused initially on the timely provision of genomic DST for individualising therapy, then reporting on clusters and strain-relatedness information to public health services. Although specific structures and processes will vary contextually, the ideal model for establishing programmes ultimately involves such systems incorporating feedback from clinicians and public health services. Clinical data can provide critical information on such aspects as the correlation of WGS findings and patient outcomes, whereas public health information is necessary for the robust interpretation of WGS findings and potential clustering.^16^ For example, two strains that are identical on WGS may be a result of local transmission, a laboratory contamination event or labelling error, or international exposure to a third common case. Differentiating between such scenarios requires public health investigation and feedback. Conversely, public health feedback can also provide information on cases which are unrecognised by WGS, such as culture-negative TB in children within a household setting. Finally, we also recognise the importance of understanding TB clustering within the context of an affected community and incorporating community voices into planning the effective use of genomic information for individual and public health benefits.^17,18^

**Table 1.** Overview of requirements for and benefits from routine whole-genome sequencing (WGS) in clinical and public health practice.

	Requirements	Benefits
Laboratory	• Technical capacity for real-time WGS	• Timely recognition of laboratory contamination
	• Long-term investment in adequate storage and sequencing infrastructure	• Reduce reliance on phenotypic drug susceptibility testing
	• Long-term investment in adequate data systems and bioinformatic expertise	
Clinical	• Simple and clear reporting from laboratories	• Optimise treatment plans and isolation requirements for drug resistance
	• Education regarding the clinical use and public health implications of genotypic findings	• Improved understanding of epidemiological context, including isolation and contact tracing requirements
	• Maintain good communication pathways with laboratories and public health	• Enhanced awareness of TB risk factors
Public health	• Capacity to coordinate timely multi-disciplinary meetings	• Increased efficiency in targeted public health responses
	• Flexible public health staff to respond to findings	• Monitoring, comparing and benchmarking program performance with standardised metrics
	• Public health database with linkage of relevant laboratory and clinical information	
	• Legal and governance framework for data sharing and linkage, as well as privacy protection	
People with TB	• Understanding the ‘added value’ of WGS for their personal care and community protection	• Better person-centred care; Individualised therapy
	• Willingness to provide information on TB history and potential contacts	• Increased confidence in accurate diagnosis and optimal treatment
Affected community	• Forum for sharing relevant WGS findings in a respectful and sensitive way	• Improved understanding of local risk factors for recent transmission
	• Education regarding the interpretation and implications of WGS	• Enhanced capacity and better-informed participation in community activities for TB risk reduction

## Implementation and impact

Information derived from WGS should be incorporated into public health programmes at various levels. Rather than being solely descriptive, a holistic integration of WGS allows for meaningful change in policy and practice from direct to strategic, which we describe as micro (individual), meso (population) and macro (policy) levels of public health (Table 2).^19^ Periodic evaluation of genomic information allows for considered reflection on optimal contextual integration at each of these levels.

**Table 2.** Levels of decision-making and examples of public health application of *M tuberculosis* whole genome sequencing (WGS). Adapted from Denholm JT, et al. Developing best practice public health standards for whole genome sequencing of *Mycobacterium tuberculosis*. Lancet Regional Health-Western Pacific. 2024;46:101014.

	Levels of decision-making	Potential implications of decision shift	Considerations for decision-making
Macro-level implications (health policy)	• Formulation of national policy and strategic plan	• Monitor program effectiveness through review of relapse/reinfection	• Undertake periodic multisectoral review to prioritise and contextualise response.
	• Monitoring and evaluation of performance and progress	• Consider whole-of-system engagement eg migration screening, labour protections	
Meso-level implications (TB programmes)	• Plan targeted interventions based on apparent transmission	• Optimise treatment plans and isolation requirements for drug resistance	• Regular and active community engagement for planning and implementing case finding and educational interventions
	• Support laboratory accreditation	• Improved understanding of epidemiological context, including isolation and contact tracing requirements	
		• Enhanced awareness of TB risk factors	
Micro-level implications (individual cases/clusters)	• Therapeutic decision-making	• Improved understanding of local risk factors for recent transmission	• Ensure equity is prioritised in responses
	• Contact investigation	• Enhanced capacity and better-informed participation in community activities for TB risk reduction	• Human rights-based approach to promoting health and reducing stigma

## Micro-level genomic data

At a micro-level, genomic data allows for rapid recognition of the potential relatedness between strains, with greater certainty than epidemiological evidence alone or previous typing methods.^20^ Identification of contexts where transmission has demonstrably occurred may be used to enhance contact investigation and to find more individuals at risk of recent infection and progression to active disease. Conversely, WGS may also find evidence that suspected transmission has not occurred, such as where several cases within a workplace are found to be unrelated and coincidental, which may put a stop to further investigation.^21,22^ WGS may also identify instances of laboratory contamination and allow unnecessary treatment to be discontinued.^23^

Although WGS clustering may increase activity and testing for those newly identified at risk, it may also allow for more focused responses and avoid the unnecessary expansion of investigations. In low-incidence settings, multiple cases occurring within a geographic area typically leads to further community testing, while in higher-incidence settings, algorithms to detect increases in background case frequency or unusual geospatial patterns or distribution may be used.^24,25^ Demonstrating that no genomic clustering exists with routine WGS may allow programmes to limit expanded contact investigation or active case-finding, avoiding unnecessary testing and treatment and the burden on people and systems.

Although rapid genotypic identification of drug resistance is of well-recognised clinical utility, from a public health perspective, it also allows for the prioritisation of responses to situations with a high risk of multidrug-resistant TB transmission. In future, it may be that other genomic factors, such as transmission or virulence determinants, will also allow for rapid response to the highest risk scenarios, particularly in resource-limited settings.^26^

## Meso-level analysis

Beyond individual cluster evaluations and contact investigation exercises, information from WGS should be regularly combined with broader epidemiological data to consider wider and emerging trends in TB transmission and geospatial distribution. Ideally, WGS clustering information would be incorporated into routine TB surveillance systems. This meso-level analysis can occur at periodic intervals and be used to consider how resources are best directed to high-impact activities. For example, genomic data may suggest closely related isolates in people from the same cultural background, geographic area or with similar occupational or recreational interests, but with no identified epidemiological connections. This scenario would support developing a contextualised approach to further investigation (such as active case-finding among identified group members) or a broader engagement to mitigate harm (such as community education programmes about access to TB services, or expanded access to preventive therapy in at-risk people).

## Macro-level analysis

At the largest scale, data from routine WGS may be used to shape TB programmatic and health service policy to be more efficient and effective. The relative value of migration screening programmes may be influenced by evidence regarding any onward transmission following arrival. Conversely, WGS data suggesting limited transmission within a jurisdiction may encourage greater weight on the identification of those with TB infection at risk of progression to support preventative therapy. Activities at this level should be supported by the identification and definition of clades of potential interest, facilitating discussion and accurate communication and allowing them to be easily identified and followed over time. The clonal nature of the *M. tuberculosis* bacterial population has been used to identify characteristic SNPs for this purpose.^27,28^

## Evaluation

Implementation of genomics for public health has frequently lacked robust measures of the impact and success of outcomes.^29^ Although a demonstration that genomic sequencing is timely and cost-effective is valuable for supporting implementation, we consider that a multimodal evaluation of its utility and effectiveness are needed in future. This evaluation should include health economic analysis, incorporating public health and patient-level costs rather than just laboratory costs. It should also include outcome measures relevant to public health programmes, such as the impact on contact investigation and/or active case finding, identification of previously unrecognised clusters, or recognition of laboratory contamination events. We also consider that a robust evaluation of the impact of WGS for TB public health programmes should include an assessment of its importance and acceptability to patients and affected community members. Such measures might include individual-level costs, or improved efficiency in community screening interventions (as discussed above), but co-design approaches should also be used to identify and promote factors that the affected communities view as being valuable. Finally, it is also appropriate to use qualitative measures of success in an evaluation of WGS, including its impact on policy and practice, and the confidence of clinicians and community members to make treatment and public health decisions based on genomic information.

Where possible, quantitative evaluation metrics for WGS should focus on factors directly relevant to programmatic TB services and their impact. While diverse epidemiological contexts may prevent the use of targets such as the proportion of cases with transmission outside of households or relapse following treatment completion, serial evaluation can provide locally relevant targets to improve services.^30^ Although the long time-horizons for progression to active disease present challenges for program evaluation, adopting a standard and specific approach to such analysis,(such as the proportion of cases clustered at 5 SNP threshold over a 2–5 year rolling average),^31^ would allow for local programmatic review of trends over time, as well as benchmarking between similar contexts.

When reviewing WGS data and cluster review, programmes should carefully avoid unnecessary potential stigmatisation in public health messages. Associating specific clusters with factors such as country of birth, geographical location or key behavioural characteristics may appear efficient for public health messaging, but harm individuals and communities by both direct trauma and reduced participation in public health actions.^32^

## Challenges and emerging concerns

Increased routine sharing of genomic data between jurisdictions and agencies has enormous potential benefits for individuals and public health services. However, given the sensitive nature of genomic data, such exchanges must address the need for security and caution, particularly in the context of the associated individual health information that may be required for optimal impact.^33^ A robust legal framework for ensuring appropriate degrees of consent and awareness of WGS is also essential, with programmes recognising increasing requests to provide such data to assist both public and private investigations (including workplace safety assessment and litigation where TB transmission is suspected). Logistically, there is also a need for increased standardisation of data-sharing platforms to reduce workload and avoid the increased risk of error. Establishing pre-existing data-sharing agreements and common platforms for WGS information will help to streamline international efforts to better understand transmission and reactivation of TB.^34^ For example, multi-jurisdictional platforms and data-sharing agreements were developed in response to COVID-19 and have been expanded for TB public health use in Australia.^35^ Such agreements should involve governments and other stakeholder organisations – and engage community voices – to achieve a balance between the benefits and burdens of data exchange. In this way we can develop genomic resources that genuinely promote health and well-being,

## CONCLUSION

The integration of WGS into routine TB health care has significant positive potential. Thoughtful application and evaluation are required to ensure that those affected by TB benefit the most. The routine use of WGS for TB would support TB elimination efforts, particularly as it expands into high-incidence settings and allows for greater understanding of TB transmission and evolution.^36^ Programmes should be encouraged to adopt common standards for bioinformatic analysis, sharing WGS information and harmonised public health responses wherever possible. This will help to maximise the common good arising from adoption of WGS technology in public health.
